# Preparation of pH-Responsive Hydrogels Based on Chondroitin Sulfate/Alginate for Oral Drug Delivery

**DOI:** 10.3390/pharmaceutics14102110

**Published:** 2022-10-02

**Authors:** Muhammad Suhail, Hamid Ullah, Quoc Lam Vu, Arshad Khan, Ming-Jun Tsai, Pao-Chu Wu

**Affiliations:** 1 School of Pharmacy, Kaohsiung Medical University, Kaohsiung City 80708, Taiwan; 2 Department of Clinical Pharmacy, Thai Nguyen University of Medicine and Pharmacy, 284 Luong Ngoc Quyen Str., Thai Nguyen City 24000, Vietnam; 3 Department of Pharmaceutics, Faculty of Pharmacy, Khawaja Fareed Campus (Railway Road), The Islamia University of Bahawalpur, Bahawalpur 63100, Pakistan; 4 Department of Neurology, China Medical University Hospital, Taichung 404, Taiwan; 5 School of Medicine, College of Medicine, China Medical University, Taichung 404, Taiwan; 6 Department of Neurology, An-Nan Hospital, China Medical University, Tainan 404, Taiwan; 7 Department of Medical Research, Kaohsiung Medical University Hospital, Kaohsiung 80708, Taiwan; 8 Drug Development and Value Creation Research Center, Kaohsiung Medical University, Kaohsiung 80708, Taiwan

**Keywords:** hydrogels, porosity, swelling, drug release, biodegradation study

## Abstract

This study investigates pH-sensitive hydrogels based on biocompatible, biodegradable polysaccharides and natural polymers such as chondroitin sulfate and alginate in combination with synthetic monomer such as acrylic acid, as controlled drug carriers. Investigations were conducted for chondroitin sulfate/alginate-*graft*-poly(acrylic acid) hydrogel in various mixing ratios of chondroitin sulfate, alginate and acrylic acid in the presence of ammonium persulfate and N′,N′-Methylene bisacrylamide. Crosslinking and loading of drug were confirmed by Fourier transform infrared spectroscopy. Thermal stability of both polymers was enhanced after crosslinking as indicated by thermogravimetric analysis and differential scanning calorimeter thermogram of developed hydrogel. Similarly, surface morphology was evaluated by scanning electron microscopy, whereas crystallinity of the polymers and developed hydrogel was investigated by powder X-ray diffraction. Furthermore, swelling and drug-release studies were investigated in acidic and basic medium of pH 1.2 and 7.4 at 37 °C, respectively. Maximum swelling and drug release were detected at pH 7.4 as compared to pH 1.2. Increased incorporation of hydrogel contents led to an increase in porosity, drug loading, and gel fraction while a reduction in sol fraction was seen. The polymer volume fraction was found to be low at pH 7.4 compared to pH 1.2, indicating a prominent and greater swelling of the prepared hydrogels at pH 7.4. Likewise, a biodegradation study revealed a slow degradation rate of the developed hydrogel. Hence, we can conclude from the results that a fabricated system of hydrogel could be used as a suitable carrier for the controlled delivery of ketorolac tromethamine.

## 1. Introduction

A great interest has been shown in the usage of natural polymers in recent years especially in physics, chemistry, pharmaceutics, and medicine. These polymers are widely used because they are environmentally friendly, low impact, and low toxicity materials. Hence, these polymers are employed by many researchers in the preparation of different drug carrier systems [1].

Ketorolac tromethamine (Kt) is a non-steroidal anti-inflammatory drug, used in the management of pain. It is a derivative of heteroaryl acetic acid and non-selective cyclooxygenase (COX) inhibitor. It is present in racemate form in the market. Most of its COX inhibitory and analgesic activity is retained in the S-isomer. The salt of Kt is tromethamine, and can be taken orally, intravenously, intramuscularly, or as a topical ophthalmic solution. The plasma half-life of Kt is 4–6 h [2]. Several doses of Kt are needed in a day in order to maintain the constant plasma concentration. However, the frequent administration of Kt leads to certain gastrointestinal complications including gastrointestinal bleeding, peptic ulceration and perforation. Hence, different drug carrier systems have been developed by a number of researchers for sustained/controlled delivery of Kt [2,3,4]. Wagh et al. developed ethyl cellulose-based micro-/nanospheres for controlled delivery of Kt [5]. Likewise, Patil et al. (2020) developed Kt-loaded chitosan nanoparticles and demonstrated Kt sustained release for 12 h [6]. Yet, a system is needed in order to overcome the adverse effects generated as a result of multiple intakes of Kt. Hence, due to diverse features including good biocompatibility, biodegradability, hydrophilicity, and low toxicity, hydrogels are reported to be the most promising carrier system for the controlled release of therapeutic agents [7].

Hydrogels are crosslinked polymeric networks, which polymerize chemically or physically, and are used widely in medicine because of their porous structure, enduring the maximum loading of therapeutic mediators due to their high swelling capability. Both synthetic and natural polymers are used for hydrogel preparation [8,9]. Swelling leads to expansion of a chain, thus promoting the release of loaded bioactive agents. Another astonishing property of hydrogels is their response to various stimuli, such as temperature, ionic strength, pH, electromagnetic field, and electro-stimulation, etc., [10]. pH-sensitive hydrogels are the most used stimuli-responsive hydrogels. They deliver the drug to the target site of the gastrointestinal tract (GI) in a controlled way by responding to the specific pH of the medium, and thus are frequently used for controlled drug delivery [11]. 

Our research work evaluated pH-sensitive hydrogels based on modified chondroitin sulfate (CS), alginate (Al), and acrylic acid (Aa). CS is a polysaccharide, natural, biodegradable, and biocompatible polymer. This natural polymer is used generally in different pharmaceutical products [12,13]. Al is a polysaccharide polymer and has various applications in food, agriculture, drug delivery, bone healing, cosmetics, paper, pharmaceutical and biomedical industries, etc., because it has the same structural resemblance to the extracellular matrix. The main benefit of Al is its ease of gelation in a warm environment [14]. Aa is a synthetic monomer, used broadly in both biomedical and pharmaceutical fields. Aa is pH-sensitive by nature because of its functional groups (COOH), which enhances its sensitivity to the external environment. Due to its high swelling capability in basic medium, Aa is employed highly in the development of pH-responsive hydrogels [15].

Here we report on the synthesis of natural polymers-based hydrogels employed for the controlled delivery of Kt. The novelty of the current drug carrier system is based on the incorporation of pH-sensitive synthetic monomer Aa with natural polymers such as CS and Al. The combination of natural polymers with synthetic monomer resulted in development of a non-toxic and mechanical strong crosslinked network of hydrogel, which prolonged the release of the Kt. CS and Al also have carboxylic acid functional groups such as Aa, which deprotonate at high pH values. Hence, the combinations of CS, Al, and Aa have increased the response of the prepared drug carrier system especially at high pH values. The developed pH-sensitive hydrogels not only sustained the release of a drug in a controlled way but also kept the drug from involvement in gastrointestinal tract (GI) degradation and vice versa. The developed hydrogels were assessed in a series of studies. 

## 2. Materials and Methods

### 2.1. Materials

Aa and Al (MW = 10,000–600,000) were purchased from Acros (Carlsbad, CA, USA). Kt was obtained from Jeedimetla, Hyderabad, Telangana, India. N′,N′-methylene bisacrylamide and ammonium persulfate were acquired from Alfa Aesar, Lancashire, UK and Showa, Tokyo, Japan, respectively. Similarly, CS (MW = 475.379 g/m) was acquired from Sigma-Aldrich (St. Louis, MO, USA). 

### 2.2. Synthesis of CS/Al-g-pAa Hydrogels

**Figure 1:**
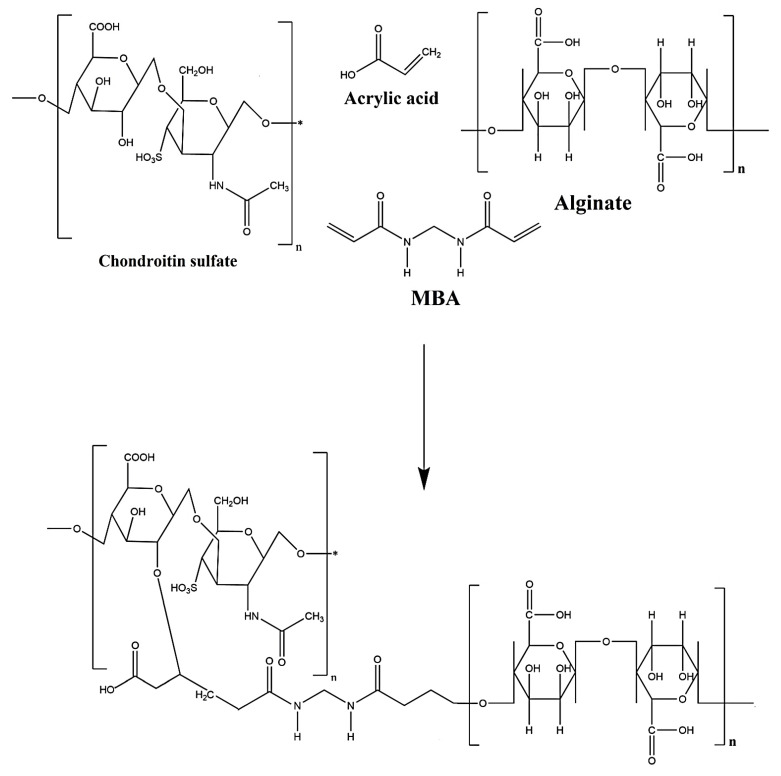
Proposed chemical structure of CS/Al-g-pAa hydrogel.

Various concentrations of CS, Al, and Aa were mixed for the fabrication of different formulations of chondroitin sulfate/alginate-*graft*-poly(acrylic acid) (CS/Al-g-pAa) hydrogels via a free radical co-polymerization technique as indicated in Table 1. An accurate amount of CS and Al was dissolved in a specific quantity of deionized distilled water and stirred with a 15 mm magnetic bar in separate beakers at 50 °C and 50 rpm. Ammonium persulfate (APS) was dissolved in distilled water and poured into the solution of Al. After a few minutes, the mixture was added into the CS solution, followed by the addition of Aa. The mixture was stirred for 15 min. N′,N′-Methylene bisacrylamide (MBA) was dissolved in water and ethanol mixture at 50 °C, and then added dropwise into the aforementioned mixture. The whole mixture was stirred continuously until a translucent solution was formed. Nitrogen gas was purged through the solution to remove dissolved oxygen. The translucent solution was poured into the glass molds, which were placed in a water bath. The temperature was fixed at 55 °C for the initial 2 h, and then increased to 65 °C for the next 23 h. The prepared gel was cut into 6 mm uniform discs and washed by a mixture of water and ethanol (50:50 *v*/*v*) to remove any entrapped unreacted content on the surface of the gel discs. The discs were kept at room temperature for 24 h and then placed in a vacuum oven at 40 °C to dry. The dried discs were subjected to a series of experiments. The proposed chemical structure of the prepared hydrogel is shown in Figure 1.

### 2.3. Fourier Transform Infrared Spectroscopy (FTIR)

FTIR is an analytical procedure employed to endorse the polymerization process needed for the preparation of hydrogels. FTIR spectrum was performed for CS, Al, Aa, the unloaded CS/Al-g-pAa hydrogel, Kt, and the loaded CS/Al-g-pAa hydrogel while using attenuated total reflectance (ATR) Bruker FTIR (Thermo Fisher Scientific, Ishioka, Japan). FTIR spectrum analysis was conducted within range of 4000–500 cm^−1^ [16]. 

### 2.4. Thermogravimetric Analysis (TGA)

Thermal stability of polymers and formulated hydrogel was investigated by TGA (PerkinElmer Simultaneous Thermal Analyzer STA 8000, (PerkinElmer Ltd., Buckinghamshire, UK). Samples of 5 mg of all unreacted ingredients and tested formulations were taken and placed in an open platinum pan attached to a microbalance. Heating of the sample was carried out within a temperature range of 40–600 °C with a heating rate of 20 °C/min. Nitrogen flow was maintained at 20 mL/min throughout the study [17].

### 2.5. Differential Scanning Calorimeter (DSC)

The glass transition temperature and both exothermic and endothermic peaks of CS, Al, and CS/Al-g-pAa hydrogel were investigated by DSC (PerkinElmer DSC 4000, Waltham, MA, USA) thermogram. Accordingly, a sample of 4 mg of unreacted CS, Al, and formulated CS/Al-g-pAa hydrogel was placed in a standard aluminum pan. DSC analysis was conducted with a heating rate of 20 °C /min within a 50–400 °C temperature range under a constant nitrogen flow of 20 mL/min.

### 2.6. Powder X-ray Diffraction (PXRD Analysis)

PXRD analysis of pure CS, Al, and CS/Al-g-pAa hydrogel was performed by X-ray diffractometer (XRD-6000 SHIMADZU, Tokyo, Japan) in order to evaluate the crystalline nature of the pure hydrogel contents and fabricated hydrogel. The samples were scanned by modifying the diffraction angles within 10° to 60° at a rate of 2° 2θ/min [18].

### 2.7. Scanning Electron Microscopy (SEM) Analysis

The surface morphology of the fabricated hydrogel was evaluated by SEM (JSM-5300 model, Jeol Ltd., Tokyo, Japan). The sample was scanned and then surface morphology was evaluated with the help of photomicrographs [19].

### 2.8. Sol-Gel Analysis

The investigation of soluble and insoluble fraction of the developed hydrogel was carried out by sol-gel fraction. Thus, a weighed hydrogel disc of each formulation was placed in a round bottom flask containing deionized distilled water and subjected to Soxhlet extraction for 12 h. Later, the extracted disc was taken out and placed at 40 °C in a vacuum oven for complete dehydration. The dried disc was then weighed again on an analytical balance [20]. Equations (1) and (2) were used for the determination of the sol and gel fraction.

(1) $$\;\mathrm{Sol}\;\mathrm{fraction}\;\%=\frac{{\;W}_{1}-{\;W}_{2}}{{\;W}_{1}}\times 100\;$$

(2) Gel fraction=100−Sol fraction

W_1_ specifies the initial weight of the dried disc of hydrogels before the extraction process, and W_2_ shows the final weight of the dried disc of hydrogels after the extraction process.

### 2.9. Porosity Study

The porosity of the developed hydrogel was evaluated by the solvent displacement method. Absolute ethanol was selected as a displacement solvent. The hydrogel disc (M_1_) of accurate weight was immersed in absolute ethanol for 72 h. After that, the disc was removed, excess ethanol was wiped, and the disc was weighed again (M_2_) [21]. Equation (3) was employed for the determination of (%) porosity.

(3) $$\left(\%\right)\;\mathrm{Porosity}\;=\frac{M_{2}-M_{1}}{\rho V}$$

ρ is the density of absolute ethanol whereas V is the swelling volume of the hydrogel disc. 

### 2.10. Swelling Studies

Swelling studies were carried out with the purpose of finding the water absorption capability of CS/Al-g-pAa hydrogel at both acidic and basic medium i.e., pH 1.2 and 7.4, respectively. The weighed hydrogel dried disc was immersed in buffer solutions of pH 1.2 and 7.4. After a specific interval of time, the disc was removed, blotted with filter paper, and then weighed again. This process was continued until an equilibrium weight of the hydrogel disc was achieved (72 h) [22]. This experiment was carried out in triplicate. Equation (4) was used for estimation of the swelling index.

(4) $$\left(q\right)=\frac{{\;L}_{2}}{L_{1}}$$

where L_1_ is the weight of the dried hydrogel disc, and L_2_ is the weight of the swelled hydrogel disc at time t.

### 2.11. Polymer Volume Fraction

Polymer volume fraction analysis was performed for the fabricated hydrogel in two different pH media, i.e., pH 1.2 and 7.4, respectively. It indicated the fraction of polymer in complete swelled form. The polymer volume fraction was estimated by using the equilibrium volume swelling (Veq) data at two different pH values (1.2 and 7.4) [23]. Polymer volume fraction was estimated by using Equation (5).

V2,s = 1/Veq(5)

V2,s indicates polymer volume fraction.

### 2.12. Drug Loading

Loading of Kt by CS/Al-g-pAa hydrogel was performed by the absorption and diffusion method. Accordingly, accurately weighed dried discs of hydrogel were submerged in a 1% drug (Kt) solution of phosphate buffer of pH 7.4 at 25 °C. The immersed discs were left in the drug solution until an equilibrium weight was obtained. After that, the loaded discs were taken out and washed with deionized distilled water to eliminate the attached drug from the surface of the hydrogel discs. The loaded hydrogel discs were kept at 40 °C in a vacuum oven for drying.

#### Quantification of Loaded Drug

The amount of loaded drug by fabricated hydrogel was determined by weight and extraction methods. In the weight method, the loaded drug was estimated by subtracting the weight of the dried unloaded disc of hydrogel from the weight of the dried loaded disc. Equation (6) was employed for the determination of the drug-loaded contents.

Drug-loaded quantity = K_L_ − K_UL_ (6)

K_L_ represents the weight of drug-loaded hydrogel discs, while K_UL_ indicates the weight of the unloaded hydrogel discs.

Another method was the extraction method. In this method, the amount of encapsulated drug by fabricated hydrogel was determined by immersing the weighed loaded hydrogel discs in 25 mL phosphate buffer solution of pH 7.4. Aliquots were taken after a specific period of time, and the medium was replaced by fresh medium of the same concentration each time. This process was continued until the entire drug was extracted completely from the hydrogel discs. The collected aliquots were then evaluated on a UV–vis-spectrophotometer (U-5100, 3 J2-0014, Tokyo, Japan) at ʎmax 280 nm in triplicate [24]. 

### 2.13. In Vitro Drug Release Studies

The release of drug from Keten (commercially available product) and developed CS/Al-g-pAa hydrogel was investigated at an acidic low pH 1.2 and basic high pH 7.4, respectively. USP dissolution apparatus-II, (Sr8plus dissolution test station, Hanson Research, Chatsworth, CA, USA) was used for performing in vitro drug release studies. A loaded weighed hydrogel disc of each formulation was placed in a buffer solution (900 mL) of both acidic and basic pH at 37 ± 0.5 °C and 50 rpm. An aliquot of 5 mL was withdrawn after a specific period of time and fresh medium of 5 mL was added back in order to keep the constant sink condition. The collected aliquots were evaluated by using a UV–vis-spectrophotometer (U-5100, 3 J2-0014, Tokyo, Japan) at ʎmax 280 nm in triplicate [25].

### 2.14. Kinetic Modeling

Zero-order, first-order, Higuchi, and Korsmeyer–Peppas models were employed in order to know the drug release mechanism of CS/Al-g-pAa hydrogels [26].

### 2.15. Biodegradation Study

Biodegradation study was conducted for CS/Al-g-pAa hydrogels in a phosphate buffer solution of pH 7.4 at 37 ± 0.5 °C. Weighed dried hydrogel discs were immersed in a phosphate buffer solution of pH 7.4 for various intervals of time, i.e., 1, 3, 5, and 7 days. After that, the discs were taken out, placed at 40 °C in a vacuum oven for drying, and then weighed again. This process was performed for 7 days [27]. The degradation rate was determined by using Equation (7).

(7) $$D\;=\frac{Z_{1}-Z_{2}}{Z_{1}}$$

D represents the degradation, Z_1_ is the dry weight of the disc while Z_2_ is the disc weight after immersion at time (t).

### 2.16. Statistical Analysis

SPSS Statistic software 22.0 (IBM Corp, Armonk, NY, USA) was employed with the purpose of presenting the results of all experiments as mean ± SD. The comparison of statistical significance was performed by Student’s *t*-test, which revealed a *p* value less than 0.05.

## 3. Results and Discussion

### 3.1. Synthesis of CS/Al-g-pAa Hydrogels

The free radical polymerization method was used for the fabrication of CS/Al-g-pAa hydrogels. In this method, free radicals of CS, Al, and Aa were formed, which were crosslinked by a crosslinker MBA in the presence of APS. The compatible structure of the fabricated hydrogel was due to the presence of MBA, which crosslinked polymers with monomer at their proper sites of action. Increase in generation of free radicals was observed as the composition of polymers and monomer was increased, which led to fast and rapid polymerization, and thus hydrogels were developed. The whole process of polymerization was carried out for 23 h, where the temperature was fixed at 55 °C for an initial 2 h, and then later enhanced to 65 °C until the entire polymerization reaction had occurred.

### 3.2. Fourier Transform Infrared Spectroscopy (FTIR)

**Figure 2:**
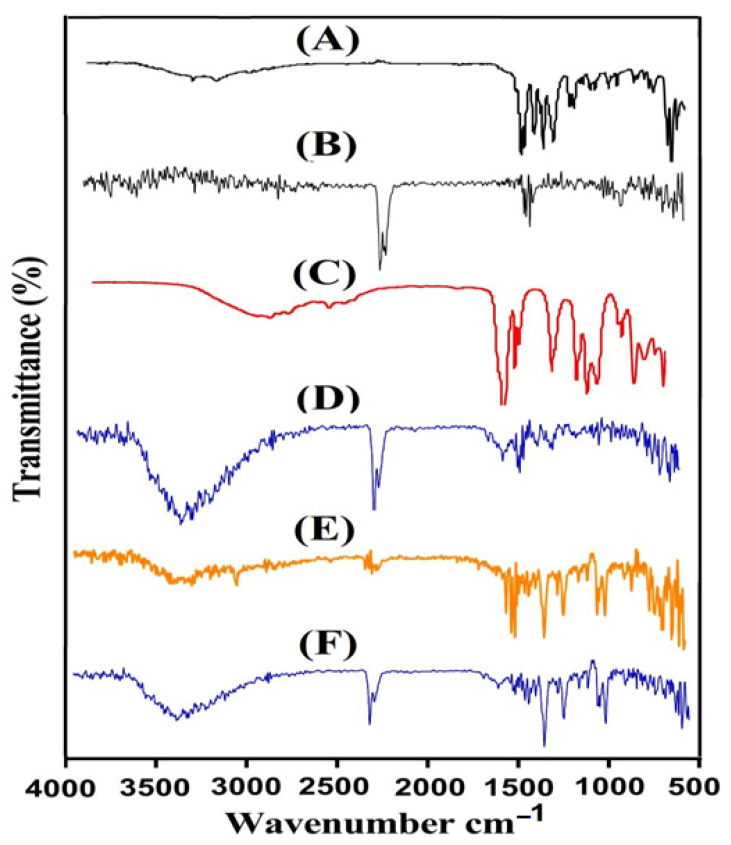
FTIR spectra of (A) CS, (B) Al, (C) Aa, (D) the unloaded CS/Al-g-pAa hydrogel, (E) Kt, and (F) the drug-loaded CS/Al-g-pAa hydrogel.

The structural arrangement of CS, Al, Aa, the unloaded CS/Al-g-pAa hydrogel, Kt, and the loaded CS/Al-g-pAa hydrogel was investigated by FTIR analysis. FTIR spectrum revealed distinctive peaks of CS (Figure 2A) within a range of 3500–3000 cm^−1^, which may be assigned to the stretching vibration of OH and N—H. The stretching vibration of the amide group was perceived by a characteristic peak at 1628 cm^−1^. C—O and OH overlapping at two different peaks, 1388 and 1430 cm^−1^, indicated the existence of a COOH group. A band at 1250 cm^−1^ indicated the existence of a sulfate group (S=O) [28]. Similarly, FTIR spectra of Al (Figure 2B) indicated asymmetric and symmetrical vibration of COO^−^ at 1627 and 1487 cm^−1^, whereas peaks at 3339 and 1256 cm^−1^ presented the stretching vibration of OH group. The corresponding peaks of C–C–C and C–O–C of the pyranic bond were depicted at 988 and 1028 cm^−1^ whereas an aliphatic C-H stretching vibration was presented by a band at 2894 cm^−1^ [29]. Likewise, the FTIR spectra of Aa as shown in Figure 2C perceived –CH_2_ and –C–C stretching vibration by peaks at 3010 and 1652 cm^−1^, while the stretching vibration of –C=O was detected by a band at 1319 cm^−1^ [30]. A fluctuation in the characteristic bands of CS, Al, and Aa was seen in unloaded CS/Al-g-pAa hydrogel (Figure 2D) due to the crosslinking and electrostatic interaction among them. The peaks of CS and Al at 1250, 1628 cm^−1^ and 1028, 1627 cm^−1^ were changed to 1290,1645 cm^−1^ and 1044, 1570 cm^−1^ bands of unloaded fabricated hydrogel. Likewise, certain bands of Aa were also modified from 1319 and 1652 cm^−1^ to 1340 and 1605 cm^−1^ peaks of fabricated hydrogel. The shifting, formation, and disappearance of bands depicted the grafting of monomer on polymers backbone, which indicated the synthesis of a new polymeric network of hydrogel. FTIR spectra of Kt (Figure 2E) revealed specific peaks at 1197, 1156, 1378, and 3368 presenting the stretching vibration of –OH, C=O, –C–N, NH_2_ and N–H, respectively. Peaks at 2152 and 800 cm^−1^ indicated C–H bending [31,32,33,34]. A slight shift was detected in the FTIR peaks of Kt after loading by developed hydrogels. The prominent peaks of Kt at 1156 and 1378 cm^−1^ were shifted to 1172 and 1390 cm^−1^ peaks of loaded CS/Al-g-pAa hydrogel (Figure 2F) demonstrated no interaction between the Kt and hydrogel contents [35].

### 3.3. Thermogravimetric Analysis (TGA)

**Figure 3:**
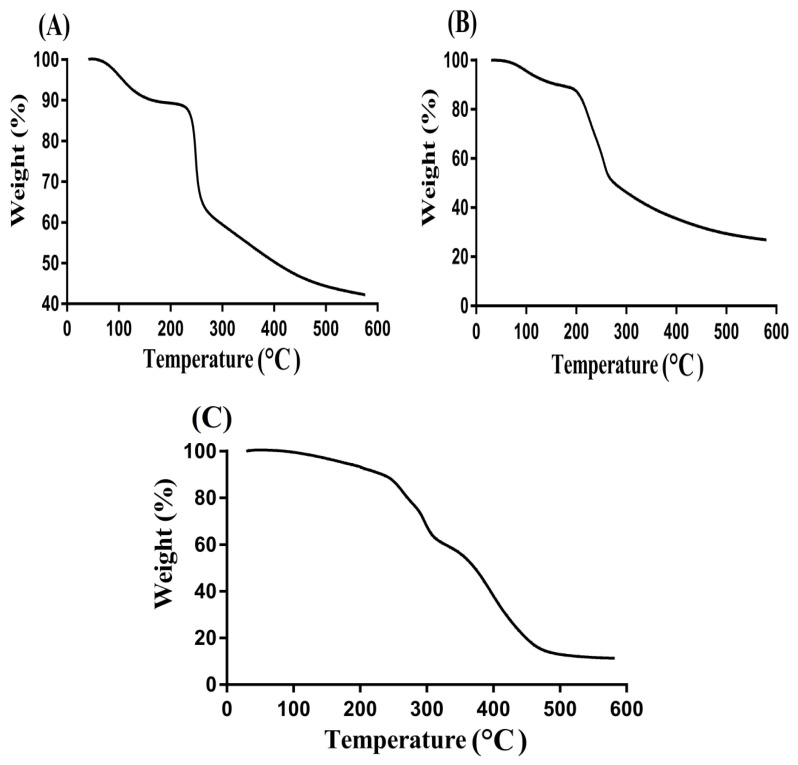
TGA of (**A**) CS, (**B**) Al, and (**C**) CS/Al-g-pAa hydrogels.

TGA was performed for CS, Al, and CS/Al-g-pAa hydrogel as shown in Figure 3. A loss of 21% in weight was seen by TGA of CS (Figure 3A) within a temperature range of 98–252 °C due to moisture loss resulting from anhydride formation by polymer chains. Onward temperature from 253 to 352 °C caused in weight loss of 28% indicated primary degradation of functional groups of the CS. Finally, degradation of CS started at 352 °C and continued up to complete degradation [36]. Similarly, pure Al indicated a 15% loss in weight as temperature approached 178 °C, as shown in Figure 3B. Due to moisture loss, a weight loss of 35% was observed further between 188 and 248 °C. Furthermore, 8% weight loss was detected with the further enhancement in temperature up to 402 °C, and onward temperature caused in Al degradation [37]. Figure 3C indicated greater thermal stability of developed CS/Al-g-pAa hydrogel as compared to CS and Al. A 37% weight loss was observed within 98–298 °C temperature range. Similarly, a loss of 23% in weight was observed as the temperature reached 400 °C. Further weight loss of fabricated hydrogels was detected with onward temperature and a 20% weight loss was seen at 480 °C. Onward temperature led to hydrogel degradation. Comparing the TGA of hydrogel contents with hydrogel formulation, we can see that thermal stability of the prepared hydrogel is higher than its contents. Thus, we can conclude that greater thermal stability of the prepared drug carrier system was due to the strong grafting and crosslinking of CS and Al with Aa [38].

### 3.4. Differential Scanning Calorimeter (DSC)

**Figure 4:**
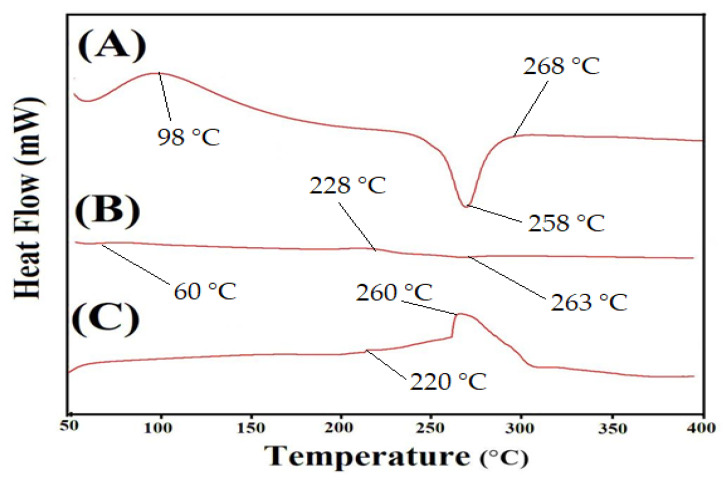
DSC of (A) CP, (B) Al, and (C) CS/Al-g-pAa hydrogel.

DSC analysis was performed for CS, Al, and CS/Al-g-pAa hydrogel (Figure 4). An endothermic peak was observed by DSC of CS (Figure 4A) within the 52–72 °C range, trailed by loss of water and other volatile substances. A strong endothermic peak at 258 °C was depicted, indicating the degradation of CS. Similarly, two exothermic peaks were detected at 98 and 268 °C. The first peak could correspond to glass transition temperature Tg, while the second peak presented oxidative degradation of CS [39]. Likewise, two minor endothermic peaks were detected at 60 and 263 °C while at 228 °C, an exothermic peak was perceived by DSC of Al (Figure 4B). The endothermic peaks indicated water loss correlated with hydrophilic groups, whereas exothermic peak indicated the dehydration and depolymerization reactions [40]. Endothermic and exothermic peaks at 220 and 260 °C were detected by the DSC of developed hydrogel (Figure 4C). A change was observed in the endothermic and exothermic peaks of both CS and Al as the endothermic peak of Al at 60 °C moved to 55 °C while the exothermic peaks of CS and Al at 228 and 182 °C were crosslinked during the polymerization reaction and observed at 220 °C in developed hydrogel. The exothermic peak of CS shifted from 268 to 260 °C, indicating the degradation of CS in its unreacted state. After crosslinking, the peak shifted and the thermal stability of CS was enhanced as shown by the DSC of prepared hydrogel. Therefore, the graphs indicated that fabricated hydrogel is more stable thermally than pure CS and Al [41].

### 3.5. Powder X-ray Diffraction (PXRD) Analysis

**Figure 5:**
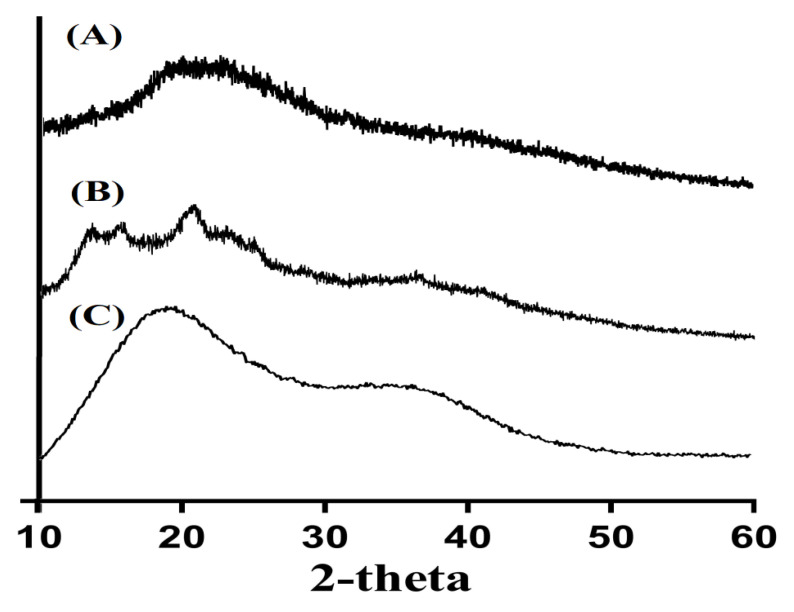
PXRD of (A) CS, (B) Al, and (C) CS/Al-g-pAa hydrogel.

PXRD was performed to evaluate the crystalline nature of CS, Al, and CS/Al-g-pAa hydrogel, as shown in Figure 5. The minor crystalline peaks of CS and higher crystalline sharp peaks of Al were observed by 2θ = 17.90°, 27.60°, and 29.80° and 2θ = 12.35°, 15.72°, 20.44° and 25.65°, respectively. These all indicated the crystalline nature of both CS and Al, as shown in Figure 5A,B. The crystallinity of CS and Al was decreased by the developed hydrogel due to the strong crosslinking of CS and Al with Aa (Figure 5C). Hence, this indicated the amorphous nature of CS/Al-g-pAa hydrogel. Generally, a decrease in the crystallinity of the reagents leads to an increase in the possibility of degradation. However, in the current study, the developed hydrogel remained stable even with a reduction in crystallinity of the CS and Al. These may be correlated with the usage of monomer Aa and crosslinker MBA. Aa was crosslinked strongly with the polymers CS and Al by MBA, due to which the possibility of hydrogel degradation was decreased. Lee et al. prepared a polymers-based hydrogel and reported a reduction in the crystallinity of hydrogel constituents by the developed hydrogel [42].

### 3.6. Scanning Electron Microscopy (SEM) Analysis

**Figure 6:**
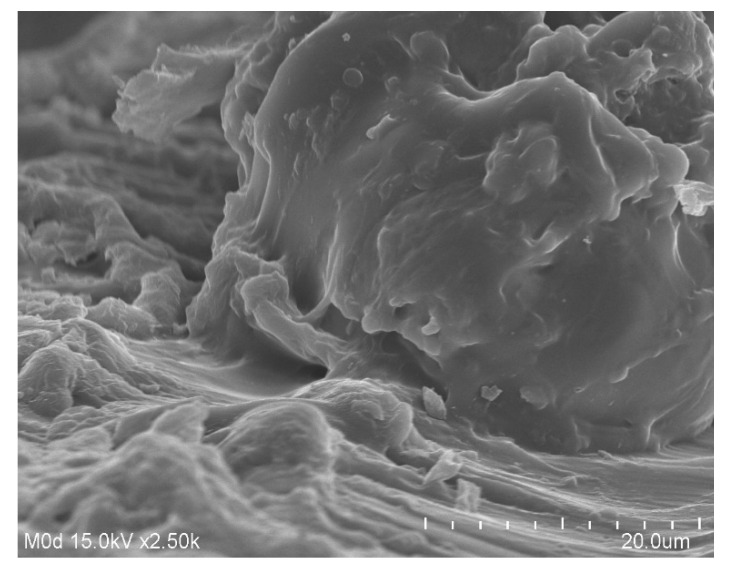
SEM of CS/Al-g-pAa hydrogel.

The surface morphology of the CS/Al-g-pAa hydrogel (Figure 6) was examined by SEM. A hard and irregular surface was revealed by the SEM, which indicated a bulk and high crosslinked network of fabricated hydrogel. Due to high crosslinking, degradation of the hydrogel was reduced while stability was enhanced. The hard and high crosslinked network of hydrogel may be attributed to the sufficient usage of MBA during the hydrogel preparation. MBA crosslinked the hydrogel contents very tightly, which led to reduction in pore size of the hydrogel, and thus a decrease in the penetration of water was observed and vice versa. Thus, high swelling, loading, drug release, and biodegradation studies demonstrated that the fabricated hydrogel network can be applied as a suitable candidate for controlled delivery of drug without losing its structural integrity [43].

### 3.7. Sol-Gel Analysis

Sol-gel analysis was performed for fabricated hydrogel to determine the insoluble crosslinked and soluble un-crosslinked fraction of the formulated hydrogels. Sol is the soluble un-crosslinked while gel is the insoluble crosslinked fraction of the fabricated hydrogel. Both sol and gel fractions were affected by different composition of CS, Al, and Aa (Table 2). The gel fraction was increased as the composition of CS and Al increased. High composition of both CS and Al led to generation of a greater number of free radicals, which further sped up the polymerization of both CS and Al with Aa, and as a result hydrogels with greater gel fraction were developed. Thus, high composition of CS and Al resulted in high gel fraction. Likewise, the gel fraction was enhanced with the increase in Aa composition. High composition of Aa led to fast polymerization reaction among hydrogel contents because free radicals were produced in a high amount with the increasing composition of Aa, and as a result hydrogels with a high gel fraction were developed. Unlike the gel fraction, a decrease in sol fraction was observed with the increase in CS, Al, and Aa composition, because there is an inverse relation between the sol and gel fraction. An increase in gel fraction leads to a decrease in sol fraction [43,44].

### 3.8. Porosity Study

**Figure 7:**
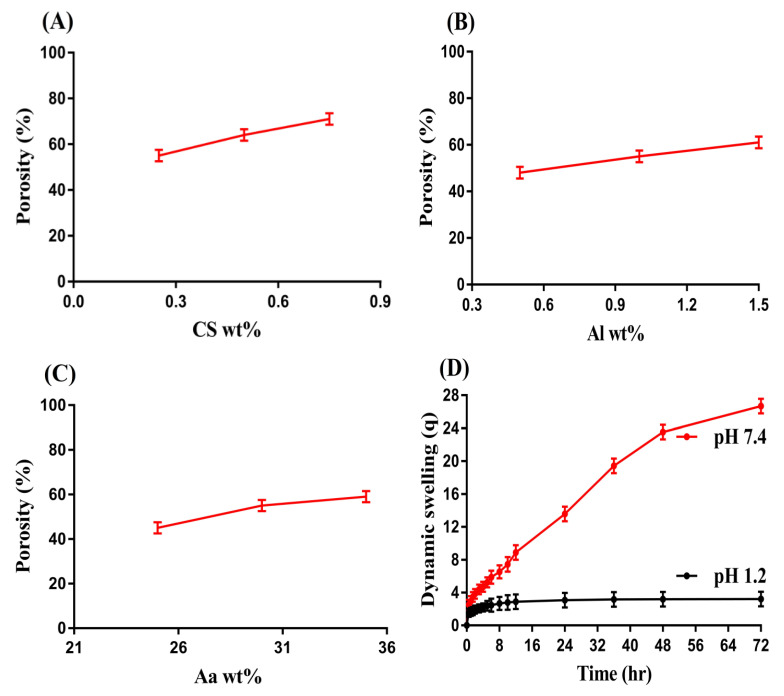
Effect of (**A**) CS, (**B**) Al, (**C**) and Aa on porosity of CS/Al-g-pAa hydrogel, and (**D**) effect of pH on dynamic swelling of CS/Al-g-pAa hydrogel.

A key role is played by porosity in both swelling and loading of drug by hydrogel. It provides channels for the penetration of solvents into hydrogel networks. Porosity is affected by the different compositions of CS, Al, and Aa as indicated in Figure 7. An increase in porosity was observed with the increasing composition of CS, AL, and Aa content. The reason may be the viscous nature of the reaction mixture. An increase in the viscosity of reaction mixture occurred as the compositions of CS, Al, and Aa increased during the polymerization process, and as a result evaporation of bubbles was constrained. This viscous mixture produced interconnected channels, which led to high porosity. Thus, an increase in porosity led to an increase in swelling and drug loading [45].

### 3.9. Swelling Studies

Swelling studies were performed for fabricated hydrogel to analyze the pH-sensitivity at two different pH media, i.e., pH 1.2 and 7.4, respectively. The swelling index of the fabricated hydrogel was found to be higher at pH 7.4 compared to pH 1.2 (Figure 7D) revealing the pH-sensitive nature of the fabricated hydrogel. The greater swelling at pH 7.4 was due to the deprotonation of the functional groups of the hydrogel contents. CS, Al, and Aa contained carboxylate (COOH) functional groups. The carboxylate groups of CS, Al, and Aa were protonated at acidic pH 1.2 and formed conjugate with counter ions through strong hydrogen bonding. Due to hydrogen bonding, charge density of the same group ions was reduced and thus almost a low swelling achieved at pH 1.2. As the pH increased from a lower (1.2) to upper (7.4) value, deprotonation of carboxylate groups of CS, Al and Aa was perceived, which led to an increase in charge density of the same charged ions. Thus, an expansion was seen in the hydrogel networks due to the generation of strong electrostatic repulsive forces. Hence, high swelling of hydrogel networks was observed [46,47].

The swelling degree of fabricated hydrogel was also affected by the different composition of CS, Al, and Aa as indicated in Table 3. An increase in swelling was detected with the enhancement in the composition of CS and Al at both pH 1.2 and 7.4, respectively. A high number of carboxylate groups was produced as the composition of CS and Al increased, which led to high charge density, and thus strong repulsive forces were produced among the same charged ions. Thus, an increase in swelling was detected with the enhancement in CS and Al composition. Similarly, the swelling index of developed hydrogel was increased as the composition of Aa increased. The reason may be associated with the generation of carboxylate ions in a high amount, which produced strong repulsive forces. These forces led to high swelling and thus expansion in hydrogel volume was observed [48,49,50].

### 3.10. Polymer Volume Fraction

Polymer volume fraction is the fraction of polymer in a swelled state of hydrogel to obtain the fraction of a polymer after the penetration of a fluid into the hydrogel networks. The polymer volume fraction of all formulations of the developed hydrogel was evaluated at both acidic and basic medium (pH 1.2 and 7.4) as indicated in Table 3. High polymer volume fraction was observed at acidic pH 1.2 as compared to basic pH 7.4. Polymer volume fraction was affected by the various composition of hydrogel content as a decrease in polymer volume was depicted with the enhancement in the composition of CS, Al, and Aa, respectively. The decrease in polymer volume fraction with the escalation in the composition of hydrogel constituents was correlated with the high swelling degree of the fabricated hydrogel contents. The low polymer volume fraction at pH 7.4 with the high at pH 1.2 indicated potential and high swelling capabilities of the prepared hydrogel at pH 7.4 [23].

### 3.11. Drug Loading

Both weight and extraction methods were employed for the estimation of loaded drug by the CS/Al-g-pAa hydrogel as indicated in Table 2. Drug loading and swelling are directly related to each other. An increase in swelling leads to an increase in drug loading [51]. Drug loading was also affected by different compositions of CS, Al, and Aa similar to swelling. A significant increase was shown in loading of drug by the fabricated hydrogel with the increasing composition of CS, Al, and Aa. The possible reason may be related to the availability of a high amount of carboxylic groups of both natural polymers and synthetic monomer, which led to greater swelling and drug loading.

### 3.12. In Vitro Drug Release Studies

**Figure 8:**
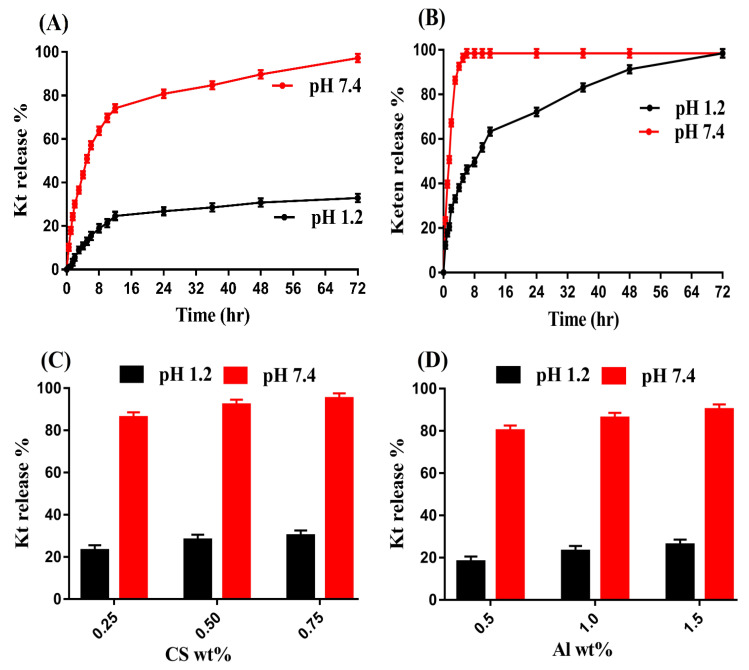

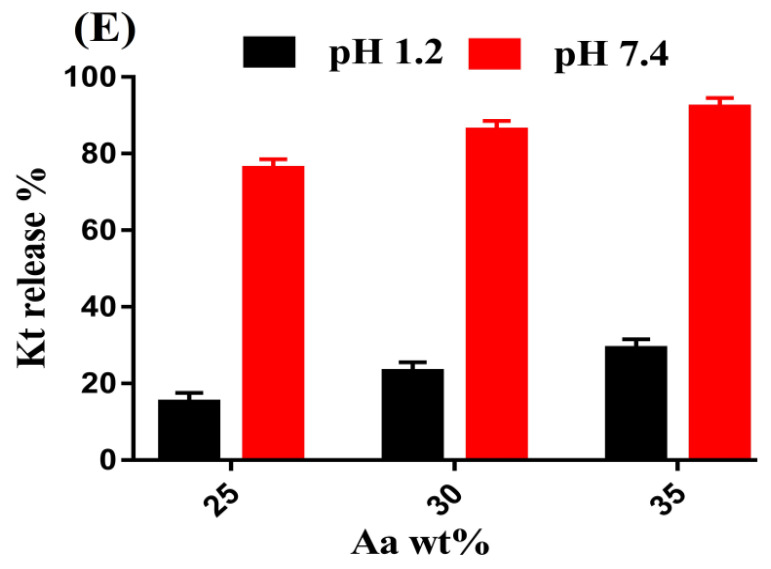
Effect of pH on drug release from (**A**) CS/Al-g-pAa hydrogel, (**B**) Keten, and Effect of (**C**) CS, (**D**) Al, and (**E**) Aa on drug release from CS/Al-g-pAa hydrogel.

Release studies were performed for all formulations of developed hydrogel and the marketed available product Keten (EVEREST, 10 mg) to know the drug release from the prepared hydrogel and Keten at pH 1.2 and 7.4, respectively. Similar to swelling, greater drug release was achieved at pH 7.4 as compared to pH 1.2 (Figure 8A) due to deprotonation of COOH groups of CS, Al, and Aa. Deprotonation of carboxylate groups led to high charged density of the same group ions, due to which electrostatic repulsive forces were generated and thus greater swelling and drug release were perceived. Contrary to pH 7.4, low release of drug was detected at pH 1.2. Due to the protonation of the functional groups of the hydrogel contents, a low drug release was observed at pH 12. Strong hydrogen bonding was formed with the counter ions, due to which charge density of the same charged ions decreased, and thus low swelling and drug release were exhibited almost at pH 1.2 [52,53]. Similar to the fabricated hydrogel, a drug release study was also conducted for the commercially available product Keten (Figure 8B) at both low and high pH values. Drug release of more than 90% was perceived within an initial 3–4 h at pH 7.4, while drug release at pH 1.2 was found at more than 88% within 48 h. Comparing the drug release of Keten and developed hydrogel, we can predict that drug release will be sustained for a long period of time by the prepared hydrogel in a controlled pattern. Hence, the developed polymeric carrier system could be used as a potential alternative to a conventional dosage form.

Drug release was increased as the composition of CS increased, as indicated in Figure 8C. Increase in generation of COOH, SO_3_ and OH functional groups of the CS occurred with the high incorporated CS content, which led to greater swelling and drug release at both pH 1.2 and 7.4 [54]. Similarly, drug release was enhanced as the composition of Al and Aa (Figure 8D,E) was enhanced and vice versa [15,49].

### 3.13. Kinetic Modeling

The chemical architecture of the reagents used in the preparation of hydrogel affected the swelling index of the prepared hydrogel. Various kinetic models including zero-order, first-order, Higuchi, and Korsmeyer–Peppas models were applied to release data in order to evaluate the mechanism of drug release from the prepared hydrogel. First-order was followed by all CS/Al-g-pAa hydrogel formulations as ‘‘r^2^” values of all other kinetic models were found less than the first-order values, as indicated in Table 4. The type of diffusion was demonstrated by ‘‘n” value. 45 ≤ n corresponds to Fickian diffusion, whereas 0.45 ≤ n ≤ 0.89 can be attributed to non-Fickian diffusion. The “n” values were obtained within a range of 0.4712–0.6308 for all CS/Al-g-pAa hydrogel formulations demonstrating non-Fickian diffusion [55,56].

### 3.14. Biodegradation Study

**Figure 9:**
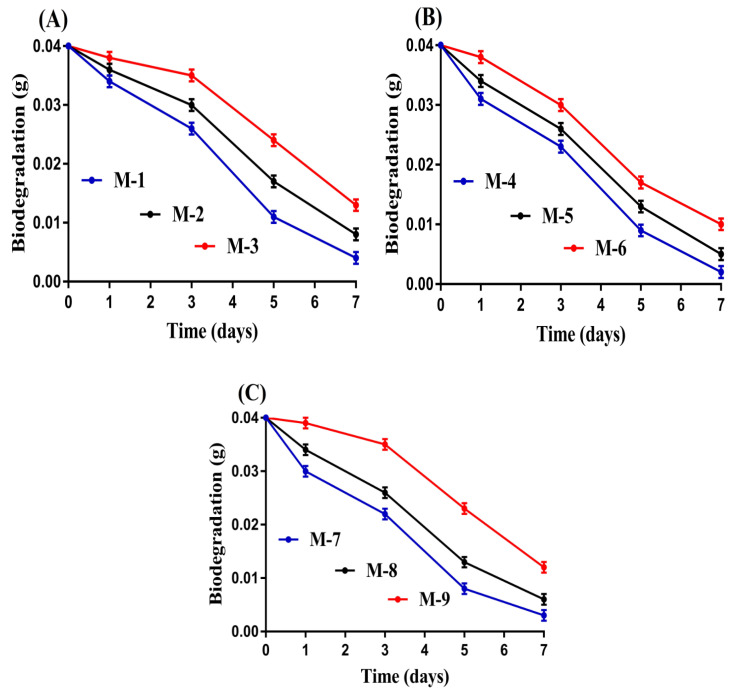
Effect of (**A**) CS, (**B**) Al, and (**C**) Aa on biodegradation of CS/Al-g-pAa hydrogel.

This study was performed to find the degradation rate of CS/Al-g-pAa hydrogel for a specific period of time. The degradation rate of the fabricated hydrogel was affected by the various compositions of the polymers and monomer, as indicated in Figure 9A–C. The degradation rate was slow, with the incorporation of high compositions of CS and Al (Figure 9A,B). The high compositions of CS and Al led to the generation of a high amount of free radicals due to the presence of a greater number of their functional groups. These free radicals caused high crosslinking and polymerization, thus a strong crosslinked hydrogel network was formed. Similarly, a slow degradation rate was seen with the increase in compositions of Aa (Figure 9C). A very slow degradation rate was depicted for Aa contents (M-9) as compared to CS and Al contents. The reason may be the usage of a high concentration of Aa, which polymerized rapidly with CS and Al in the presence of crosslinker. Hence, we can say that the degradation rate becomes slow with the incorporation of high compositions of CS, Al, and Aa. Mohamed et al. fabricated hydrogels of chitosan and polyvinyl alcohol and stated a slow degradation rate for the prepared hydrogel with the high incorporated hydrogel constituents [57], which further supports our findings.

### 3.15. Comparison of Kt-Loaded CS/Al-g-pAa Hydrogels with Other Kt Delivery Systems

Free radical polymerization is the most common method applied for the preparation of hydrogel because no specific pressure and temperature is required for this technique. The process of polymerization and crosslinking occurs very rapidly among the hydrogel contents, which leads to fast gel formation [58].

A comparison of Kt-loaded CS/Al-g-pAa hydrogels with other Kt delivery systems is made on the basis of maximum % drug release and time for maximum % drug release, as shown in Table 5. The data of Table 5 show that release of the Kt was sustained for a prolonged time by the prepared hydrogels as compared to other drug delivery systems. The benefit of the prepared pH-sensitive hydrogel is not only limited to control release of Kt but also protects the GI, especially the stomach, from the adverse effects of the Kt. Additionally; Kt is also protected from the acidic environment of the stomach. Hence, the prepared hydrogel networks could be considered as a promising carrier for the controlled delivery of drugs.

## 4. Conclusions

The natural polymers-based hydrogels were formed successfully by the crosslinking free radical polymerization technique. A very low swelling and drug release were observed at acidic medium while higher at basic medium, demonstrating Ketorolac tromethamine controlled release profile. The maximum swelling and drug release in basic medium were due to the deprotonation of functional groups of polymers and monomer. Increased incorporation of chondroitin sulfate and alginic acid with acrylic acid led to maximum swelling, drug release profile, porosity, drug loading, and gel fraction whereas sol fraction was decreased. Increase in porosity led to maximum swelling and drug loading because the pore size of the developed hydrogel was increased, due to which maximum water penetration into the hydrogel networks occurred and as a result high swelling and drug loading were detected ultimately. Similarly, all formulations of CS/Al-g-pAa hydrogel followed the first order of kinetics where “n” values demonstrated non-Fickian diffusion. A biodegradation study reported a slow degradation rate for all formulations of the developed hydrogel. FTIR presented the structural arrangement and preparation of the polymeric hydrogel. Likewise, TGA and DSC were performed for polymers and developed hydrogel and predicted greater thermal stability of the fabricated hydrogel as compared to pure contents, i.e., chondroitin sulfate and alginic acid. The high thermal stability of the fabricated hydrogel was the increase in the thermal stability of the polymers after crosslinking and polymerization process. SEM indicated a hard surface of hydrogel presented a good compatibility of the formulated hydrogel. The crystallinity of chondroitin sulfate and alginate was decreased by developed hydrogel as revealed by PXRD analysis. Regarding the results of the studies, we demonstrated that the formulated hydrogels are not only limited to control release of Ketorolac tromethamine but could be applied for drugs associated with stomach acidity complications.

## Figures and Tables

**Table 1:** Chemical composition for formulation of CS/Al-g-pAa hydrogels.

F. Code	Polymer CS g/100	Polymer Al g/100 g	Monomer Aa g/100 g	Initiator APS g/100 g	Cross-Linker MBA g/100 g
M-1	0.25	1.0	30	0.5	0.5
M-2	0.50	1.0	30	0.5	0.5
M-3	0.75	1.0	30	0.5	0.5
M-4	0.25	0.5	30	0.5	0.5
M-5	0.25	1.0	30	0.5	0.5
M-6	0.25	1.5	30	0.5	0.5
M-7	0.25	1.0	25	0.5	0.5
M-8	0.25	1.0	30	0.5	0.5
M-9	0.25	1.0	35	0.5	0.5

**Table 2:** Sol-gel analysis and drug loading of CS/Al-g-pAa hydrogels.

Formulation Code	Sol Fraction %	Gel Fraction %	Drug-Loaded (mg)/500 mg of Dry Gel	
			Weight Method	Extraction Method
M-1	08.20	91.80	394.50 ± 0.88	392.64 ± 1.09
M-2	07.03	92.97	416.82 ± 0.97	415.54 ± 1.14
M-3	06.64	93.36	435.74 ± 1.08	433.42 ± 1.25
M-4	09.84	90.16	339.53 ± 1.12	338.92 ± 0.78
M-5	08.20	91.80	394.50 ± 0.88	392.64 ± 1.09
M-6	07.12	92.88	407.92 ± 0.92	405.62 ± 0.98
M-7	10.74	89.26	315.60 ± 1.10	314.08 ± 1.05
M-8	08.20	91.80	394.50 ± 0.88	392.64 ± 1.09
M-9	06.90	93.10	432.82 ± 1.04	431.69 ± 0.89

**Table 3:** Dynamic swelling and polymer volume fraction of CS/Al-g-pAa hydrogels.

Formulation Code	Dynamic Swelling up to 72 h		Polymer Volume Fraction	
	pH 1.2	pH 7.4	pH 1.2	pH 7.4
M-1	2.90 ± 0.19	22.62 ± 0.32	0.344	0.044
M-2	3.12 ± 0.21	25.10 ± 0.23	0.320	0.039
M-3	3.21 ± 0.17	26.68 ± 0.18	0.311	0.037
M-4	2.58 ± 0.28	19.39 ± 0.14	0.387	0.051
M-5	2.90 ± 0.19	22.62 ± 0.32	0.344	0.044
M-6	3.06 ± 0.22	24.42 ± 0.17	0.326	0.040
M-7	2.50 ± 0.31	17.14 ± 0.20	0.400	0.058
M-8	2.90 ± 0.19	22.62 ± 0.32	0.344	0.044
M-9	3.14 ± 0.25	25.29 ± 0.19	0.318	0.039

**Table 4:** Kinetic modeling release of Kt from CS/Al-g-pAa hydrogels.

F. Code	Zero Order r^2^	First Order r^2^	Higuchi r^2^	Korsmeyer–Peppas	
				r^2^	n
M-1	0.8886	0.9822	0.9058	0.9438	0.5941
M-2	0.9342	0.9888	0.9867	0.9568	0.5555
M-3	0.8954	0.9856	0.9790	0.9762	0.4712
M-4	0.9079	0.9937	0.9824	0.9550	0.6308
M-5	0.8886	0.9822	0.9058	0.9438	0.5941
M-6	0.8383	0.9808	0.9475	0.9445	0.5519
M-7	0.8939	0.9876	0.9783	0.9577	0.6244
M-8	0.8886	0.9822	0.9058	0.9438	0.5941
M-9	0.8700	0.9940	0.9654	0.9594	0.5393

**Table 5:** Comparison of Kt-loaded CS/Al-g-pAa hydrogels with other Kt delivery systems.

S. No.	Formulation	Maximum % Drug Release	Time for Maximum % Drug Release	Reference
1	Albumin based microspheres	100	24 h	[3]
2	Ethyl cellulose based micro/nanospheres	58	12 h	[5]
3	Kt-loaded chitosan based nanoparticles	98	12 h	[6]
4	Kt-loaded films of Kollidon SR	100	8 h	[59]
5	CS/Al-g-pAa hydrogels	98	72 h	Current study
